# Delivery of Toxins and Effectors by Bacterial Membrane Vesicles

**DOI:** 10.3390/toxins13120845

**Published:** 2021-11-26

**Authors:** Adrian Macion, Agnieszka Wyszyńska, Renata Godlewska

**Affiliations:** Department of Bacterial Genetics, Institute of Microbiology, Faculty of Biology, University of Warsaw, Miecznikowa 1, 02-096 Warsaw, Poland; az.macion@student.uw.edu.pl (A.M.); ak.wyszynska@uw.edu.pl (A.W.)

**Keywords:** membrane vesicle, virulence factors, secretion systems, pathogenesis, bacterial toxins

## Abstract

Pathogenic bacteria interact with cells of their host via many factors. The surface components, i.e., adhesins, lipoproteins, LPS and glycoconjugates, are particularly important in the initial stages of colonization. They enable adhesion and multiplication, as well as the formation of biofilms. In contrast, virulence factors such as invasins and toxins act quickly to damage host cells, causing tissue destruction and, consequently, organ dysfunction. These proteins must be exported from the bacterium and delivered to the host cell in order to function effectively. Bacteria have developed a number of one- and two-step secretion systems to transport their proteins to target cells. Recently, several authors have postulated the existence of another transport system (sometimes called “secretion system type zero”), which utilizes extracellular structures, namely membrane vesicles (MVs). This review examines the role of MVs as transporters of virulence factors and the interaction of toxin-containing vesicles and other protein effectors with different human cell types. We focus on the unique ability of vesicles to cross the blood–brain barrier and deliver protein effectors from intestinal or oral bacteria to the central nervous system.

## 1. Introduction

The first report of bacterial membrane vesicles appeared in the mid-twentieth century [1]. In this study, the protein exotoxin secreted by *Vibrio cholerae* was shown to be resistant to proteases. Transmission electron microscope (TEM) analysis suggested that this exotoxin is located within spherical structures containing components of the bacterial cell envelope. These structures, detected in cell-free supernatants obtained from liquid bacterial cultures in the exponential growth phase [2], were named membrane vesicles (MVs).

As enveloped structures, MVs have the characteristics of vectors that enable the transport of substances highly sensitive to environmental conditions. They protect proteins enclosed in their lumen against enzymatic decomposition, degradation related to low or high pH and oxidative stress conditions. Therefore, it is not surprising that, in addition to proteins acquiring of nutrients from the environment, pathogenic bacteria also use MVs to transport toxins that directly affect host cells and enzymes promoting bacterial colonization, facilitating the disruption of infected tissues and spreading of infection in the host. We provide examples of the best characterized bacterial virulence factors associated with MVs in Table 1. The enrichment of certain proteins in MVs, at a higher concentration than found in bacteria, suggests a degree of specification for MVs in toxic activity, polymer decomposition, antibiotic inactivation or metal ion sequestration. The small size of MVs (ranging from 20–250 nm in diameter) [3] permits them to overcome epithelial barriers, such as the gut–blood barrier (GBB), and enter tissues that are not colonized by the bacteria producing them. The presence of surface antigens allows MVs to interact with cells of the host immune system, so that virulence factors they transport can modulate (induce or inhibit) the immune response. MVs can also act as “traps” for antibodies circulating in the inhabited tissue, or for bacteriophages in the natural environment. The great versatility of vesicles is the result of variation in their structure and composition. The secretion of active factors in this form is one of the most complex and diverse mechanisms of bacterial interaction with the environment and other cells [4].

## 2. Structure of Membrane Vesicles (MVs) and Mechanisms of Secretion

**Figure 1:**
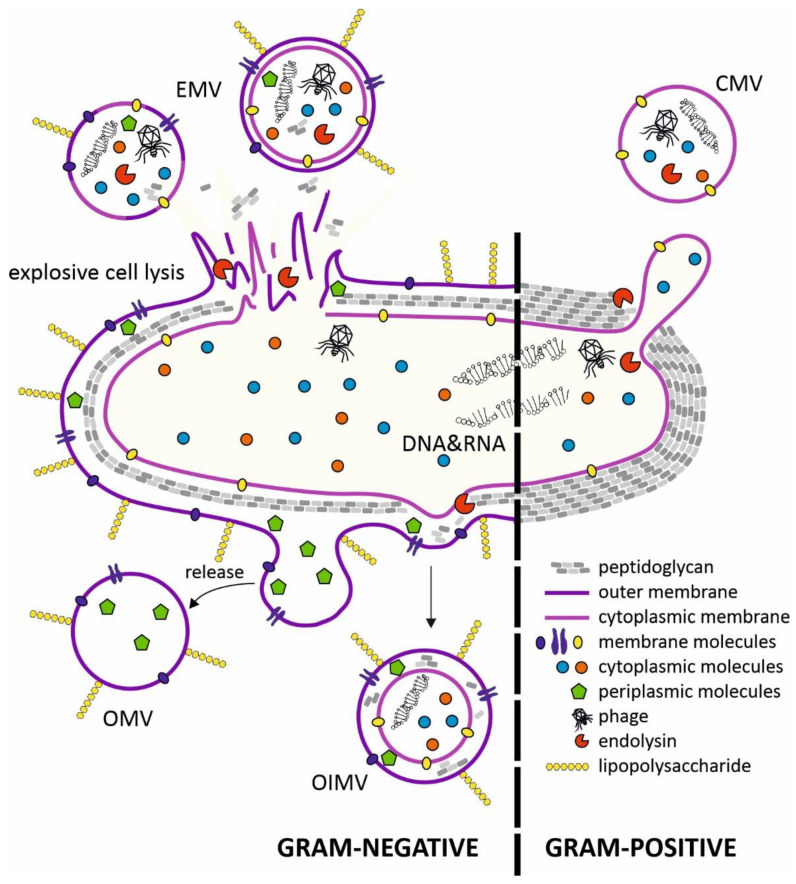
Mechanisms of bacterial membrane vesicle formation. In gram-negative bacteria, membrane vesicles are produced through membrane blebbing or explosive cell lysis triggered by phage-derived endolysins. Endolysins participate in the formation of cytoplasmic membrane vesicles (CMVs) in Gram-positive bacteria. The cytoplasmic membrane protrudes through holes in the peptidoglycan degraded by phage-derived endolysins. The contents of the membrane vesicles depends on the route of their formation. EMV—explosive membrane vesicle; OIMV—outer-inner membrane vesicle; OMV—outer membrane vesicle; CMV—cytoplasmic membrane vesicle.

The production of MVs (both extracellular and intracellular) has been observed in organisms from all three domains of life [5]. Research on bacterial vesicles has been ongoing for over 60 years, but the mechanisms of their biogenesis are still not fully understood. Several vesicle types have been described in Gram-negative and Gram-positive bacteria. The MVs exhibit the membrane features of the originating bacteria and thus could indicate the nature of their cargos, such as proteins and nucleic acids (Figure 1).

OMVs (outer-membrane vesicles) produced by Gram-negative bacteria consist of blebs of bacterial outer membrane containing transmembrane proteins and LPS, with extracellular DNA (eDNA) exposed on the surface of OMVs, with periplasmic content packaged in the lumen of the vesicle. OMVs are produced by many species of pathogenic bacteria, including *Neisseria meningitidis*, *Helicobacter pylori*, *Escherichia coli* (EHEC) and *Salmonella* spp. [6]. Increased secretion of OMVs usually occurs under stressful conditions, and is accompanied with the accumulation of misfolded proteins in the periplasm. According to one MV biogenesis model, the pressure of these defective proteins on the inner surface of the OM is responsible for bulging of the membrane and its detachment from the cell in the form of vesicles [7].

Outer-inner membrane vesicles (OIMVs) are double-membrane structures and were first observed in cultures of *Pseudomonas aeruginosa* [8]. The outer membrane and inner membrane are separated with a thin layer of periplasm with degraded fragments of peptidoglycan. The production of OIMVs are induced in stressful or adverse situations. Cytoplasm present in the lumen of these vesicles contains proteins and also fragments of DNA derived from the chromosome or plasmids and ATP [8].

Vesicles containing cytoplasm are also produced by Gram-positive bacteria. The release of CMVs (cytoplasmic membrane vesicles) requires local peptidoglycan degradation by internal or external lytic enzymes (digesting both the glycan backbone and peptide bonds in the amino acid chains) [9]. CMV production has been observed in several model Gram-positive bacteria, including *Bacillus subtilis* [10], *Bacillus anthracis* and *Staphylococcus aureus* [11,12].

The last membrane vesicle type is EMVs (explosive membrane vesicles), which are the most diverse in terms of structure. They arise spontaneously during bacterial cell lysis. Fragments of membrane, together with the outflow of cytoplasm (also periplasm in the case of Gram-negative bacteria), create spherical membrane structures in the environment. Thus, the process of EMV “assembly” is cell-independent and spontaneous, so bacteria are unable to control the content of these vesicles. As a result, each lysing cell produces MVs that differ in size, composition and function. This process has been described in *P. aeruginosa* biofilms, where deeply located cells subjected to hypoxia, nutrient deficiency and activation of the SOS system are autolysed through the activity of endolysins, and type R and F pyocins [13]. EMVs released in this way are an important factor in the virulence of pathogenic *P. aeruginosa* strains. As a component of the biofilm matrix, they bind eDNA and polysaccharides, and also bacteria via surface adhesins, which stiffens this structure [14].

Several recent reviews describe the composition and biogenesis of bacterial membrane vesicles [6,7,15,16,17]. In this article we present the latest data concerning interactions between MVs and selected human cell types.

## 3. Effects of Membrane Vesicles on Human Cells

Bacteria possess several mechanisms for the secretion of effector proteins, such as the multi-subunit T3SS (Type 3 Secretion System), T4SS and T6SS, which deliver bacterial proteins to host cells [18]. The exported proteins perform a variety of important functions, which include modulating the activity of intracellular signaling pathways, changing the structure of the cytoskeleton, and damaging mitochondria or genomic DNA [19]. In addition, membrane vesicles originating from bacterial cells can transport biologically active toxins, adhesins and factors that modulate the activity of the host immune system [20,21,22]. In the case of Gram-negative bacteria, another important component of OMVs is LPS (lipopolysaccharide). Therefore, bacterial MVs are an important source of effectors that can have a dramatic impact on the cells of a eukaryotic host. Adhesins present on the vesicle surface recognize appropriate receptors on the host cell, which enables targeting and delivery of MVs.

There are multiple ways in which MVs can enter host cells. Direct vesicle-cell fusion takes place at special membrane domains, the so-called lipid rafts, which are characterized by a high content of cholesterol causing increased rigidity. This process can also require the presence of receptor protein complexes (on the cell surface) and cytoskeleton remodeling (at the cytoplasmic face) [16].

The content of MVs enter eukaryotic cells through absorption and endocytosis. It has been shown that the absorption of such vesicles is not limited to specialized phagocytes. PAMP (pathogen associated molecular patterns) associated with the vesicle membrane are a target for antibodies causing the titration of immunoglobulins, which decreases binding to bacterial cells. The host organism attacked by the pathogen is an environment full of stressors for bacterial cells. Besides the systems of innate or acquired immunity, these include a raised temperature compared to the external environment and a dearth of iron caused by ion-sequestering host proteins, which force pathogenic bacteria to produce various fractions of membrane vesicles—e.g., vesicles with siderophores [23].

One of the most extensively studied functions of membrane vesicles is their participation in the intracellular transfer of toxins or proteins modulating defense responses. The immunotoxins most frequently secreted in this manner are peptidoglycan fragments. These are recognized by the immune system receptors NOD1 and TLR2, and this stimulates inflammation [24]. Numerous studies have shown that most known pathogens produce OMVs [3,25,26]. This thesis is supported by analyses of infected tissues as well as morphological and biochemical evidence. Vesicles are also found beyond the bounds of the local bacterial infection.

Every interaction between membrane vesicle and host cell starts with contact and absorption. This process is caused by micropinocytosis, clathrin or caveolin-mediated endocytosis, as well as by membrane fusion, which can involve lipid rafts.

Vesicles can also be phagocytized by cells of the immune system (macrophages, neutrophils and dendritic cells) or epithelial cells due to the presence of effector proteins in the vesicle membrane that interact with eukaryotic cell receptors and activate phagocytic pathways. Inside the cell, vesicles fuse with the early endosome membrane, and subsequently induce the breakdown of the endosome-lysosome complex, which releases its acidic lumenal contents, including lytic enzymes. Active bacterial factors that show resistance to lysosomal degradation enter the cytoplasm and can interact with the appropriate molecular targets. Where vesicles enter an actively migrating cell (e.g., a dendritic cell) and stable conditions are maintained inside the vesicle, the cell’s reaction to toxins carried by a certain fraction of the MVs might be delayed. If this reaction is associated with necrosis, the intact MV fraction is released upon cell lysis, and can affect other cells in the surrounding tissues [27].

Besides the direct influence of MVs on the first host cell each vesicle contacts, the process of transcytosis has also been observed. Within the Peyer’s patches (elements of the lymphoid tissue in the intestinal mucosa) there are M cells that differ morphologically from enterocytes, having a thinner glycocalyx and mucus layer, and less developed apical microvilli. M cells possess the unique ability to transfer antigens from the intestinal lumen to the connective tissue layer under the epithelium, where they contact elements of the immune system. The interaction and binding of vesicles to the M cell is enabled by the presence of PAMP in the MV membrane. The vesicle enclosed in the endosome is transported to the basolateral space, which, thanks to pocket formation, ensures interaction of the MVs with cells of the immune system (e.g., B cells, neutrophils, macrophages) [28].

### 3.1. Effects of Membrane Vesicles on Cells of the Intestinal Epithelium

The digestive tract is a habitat for a huge number of bacteria, which can be divided into three groups: beneficial (*Bifidobacterium* and *Lactobacillus*), opportunistic (*Bacteroides*, *Eubacterium* and *Enterobacteriaceae*) and pathogenic (*Clostridium*, *Staphylococcus*, *Pseudomonas* and pathogenic *E. coli*). In healthy individuals, these groups remain in a state of biological equilibrium and they interact with each other (intra- and inter-species), and with the host tissues by secreting various protein effectors. Apart from the well characterized classical bacterial secretion systems, there is growing evidence for the participation of membrane vesicles in this process. Numerous studies have investigated membrane vesicles of pathogenic bacteria and their role in transporting virulence factors and toxins into host cells. It has even been proposed to call the OMV a secretion system type 0 [29]. Several reports have demonstrated that pathogenic bacteria utilize different strategies for the transmission of virulence factors via membrane vesicles. A recent review by Rueter and Bielaszewska focused on OMVs produced by pathogenic *E. coli* strains [30].

Vesicles of ETEC (enterotoxigenic *E. coli*) contain heat-labile enterotoxin (LT)—a major virulence factor which disturbs the electrolyte balance in the host by causing the efflux of water and electrolytes from epithelial cells into the lumen of the intestine. OMV-mediated trafficking is thought to be the main route of LT export from the bacterial cell because over 95% of toxins are associated with these vesicles through an interaction with LPS [31]. LT recognizes and binds GM1 receptors present in lipid rafts, so endocytosis of LT-carrying OMVs depends on these cholesterol-rich membrane domains [32].

Another heat-labile enterotoxin, cholera toxin (CT), the major virulence factor of pathogenic strains of *Vibrio cholerae*, is delivered to eukaryotic cells by a slightly different mechanism. Multiple strains of *V. cholerae* transport this toxin via a type II secretion system (T2S) [33,34,35], but CT is also associated with OMVs [36]. Rasti and colleagues recently demonstrated that CT produced by *V. cholerae* strain 569B, in contrast to LT, is localized within the lumen of these vesicles and as a consequence OMV internalization is GM1 independent [37]. Horstman and Kuehn have suggested that vesicles without surface localized toxins can use other external proteins, e.g., OmpA, to mediate interactions with eukaryotic cells [31,38]. However, the localization of CT in OMVs appears to depend on the *Vibrio* strain because Chatterjee and Chaudhuri identified the toxin on the OMV surface. This is most likely due to variations in the structure of the LPS O-antigen [36].

Besides LT, other *E. coli* toxins are also found in OMVs. Kunsmann and co-workers characterized OMVs of *E. coli* O104:H4. This highly virulent strain was the cause of the 2011 European diarrheal outbreak and has features of two types of pathogenic *E. coli*: enterohemorrhagic (EHEC) and enteroaggregative (EAEC). OMVs of *E. coli* O104:H4 contain the major virulence factor Shiga toxin (Stx) 2a, most of which is found inside the vesicles with only a small fraction bound to the membrane. Vesicles carrying toxin Stx2a were found to penetrate epithelial cells via dynamin-dependent endocytosis, possibly mediated by clathrin [21]. Such a mechanism makes the action of Stx2a independent of the presence of globotriaosylceramide (Gb3), which is a receptor for cellular binding and internalization of free Stx2a. This is of great importance for the pathogenesis of strain O104:H4 because it allows the toxin to be delivered to the Gb3-negative human colon epithelium, which may be crucial for its systemic spread into the kidneys during hemolytic-uremic syndrome (HUS) [21]. On the other hand, Bielaszewska and colleagues showed that Gb3-negative cells cannot be intoxicated by Stx2a after MV delivery. The Gb3 serves not only as a receptor, but is also important for retrograde trafficking and cytosolic release of Stx2a, even when it is transported by MVs [39].

The cytolethal distending toxin (CDT) expressed by *Campylobacter jejuni* is another notable enteropathogen toxin transported by OMVs. CDT causes DNA damage (double-strand breaks) resulting in cell cycle arrest at the G2/M phase [40,41]. Elmi et al. showed that internalization of CDT-containing OMVs occurs via lipid raft-dependent endocytosis [42].

Thus, enteropathogenic bacteria use membrane vesicles to transport their toxins. These proteins are located in the lumen of the vesicles (CT and Stx2a of *E. coli* O104:H4), associated with their membrane (LT of *E. coli*) or are present on their surface. The binding of toxin to the vesicle membrane may determine the manner of vesicle internalization by host cells. In the case of heat-labile enterotoxin (LT), the interaction of toxin on the OMV surface with the GM1 receptor is a signal to initiate internalization, whereas localization of the toxin within the vesicle permits its delivery to host cells regardless of the presence of an appropriate cell surface receptor.

In addition to toxins, membrane vesicles of enteropathogens also contain other important proteins in the pathogenesis process. One such protein is the HtrA protease found in *C. jejuni*, *H. pylori*, *V. cholerae* and *E. coli* vesicles [43,44,45]. HtrA is a key component of the extracytoplasmic protein quality control system. Its proper functioning is essential for the survival of bacterial cells under stressful conditions, and in the case of pathogenic strains it often determines their virulence. This protease is translocated across the inner membrane to the periplasm by a Sec-dependent system. Despite considerable evidence for the presence of an extracellular HtrA fraction, no signals enabling transport across the outer membrane via known secretory systems (transporters type I–VII) have been identified in this protein [46,47]. Therefore, it is postulated that HtrA is transported by OMVs which are continuously shed by Gram-negative bacteria. The mechanism by which this protein is released from the vesicles is unknown. HtrA cleaves proteins that form junctions between epithelial cells, such as occludin and claudin-8 in tight junctions (TJ), and E-cadherin in adjacent junctions (AJ). Proteolysis of these proteins leads to disruption of the epithelial barrier, which permits the migration of bacteria through the cell monolayer and access to basolateral surfaces and deeper tissues [48,49,50,51].

In conclusion, the transport of virulence factors and protein effectors via membrane vesicles represents an alternative to classical secretion systems for the export of proteins from enteropathogenic bacteria. Vesicles provide protection to their cargo proteins, while mediating their transport over long distances and delivery to different host cell types through a toxin-independent mechanism, which undoubtedly increases the competitiveness and effectiveness of bacterial pathogens.

### 3.2. Effects on the Blood-Brain Barrier

The blood-brain barrier (BBB) is responsible for maintaining homeostasis of the central nervous system (CNS). Its basic structural elements are brain microvascular endothelial cells, astrocytes and pericytes. Maintenance of the BBB is dependent on the tight junctions occurring on the surfaces of apical membranes of endothelial cells. Tight junctions are composed of a complex of transcellular proteins: occludins, claudins and junctional adhesion molecules, bound to the actin cytoskeleton by a group of cytoplasmic proteins called “zonula occludens”. The BBB is, therefore, a physical barrier that protects the CNS from the potential negative effects of substances in the bloodstream [52,53]. A number of studies have indicated that a leaky BBB contributes to brain inflammation, the prolonged persistence of which may result in neurodegenerative disorders such as Alzheimer’s disease.

There is growing evidence that bacterial extracellular vesicles (EVs) produced by members of the intestinal or oral microbiota participate in the formation of inflammation in the CNS [54,55,56]. The mechanisms by which EVs cross the intestinal epithelial barrier have been fairly well characterized. The translocation of vesicles can occur by macropinocytosis, clathrin or caveolin-mediated endocytosis, as well as by membrane fusion which may involve lipid rafts [57]. An intracellular pathway has been proposed for OMVs from *C. jejuni*, which cleave E-cadherin and occludin [45]. In the case of the oral microbiota, gingivitis and periodontitis are factors that contribute to the entry of bacteria, and thus also OMVs, into the bloodstream. However, transient bacteremia is observed even in healthy people, e.g., after tooth extraction or brushing.

It has recently been demonstrated that bacterial EVs reach the brain after entering the systemic circulation. Wei and colleagues showed that PKH26-labeled extracellular vesicles injected into the tail vein of mice were subsequently detected in the hippocampus [56]. However, the mechanism by which these vesicles crossed the BBB is unclear. Using intravital imaging of mice, Ha and co-workers showed that the transport of OMVs to cortex microglial cells is mediated by meningeal macrophages [58]. Other studies indicate the role of the vagus nerve in this process. Following oral administration of fluorescein-labeled OMVs or LPS from *Paenalcaligenes hominis*, significantly more of the former was found to be accumulated in the hippocampus. On the other hand, vagotomy blocked only the transport of the fluorescein-conjugated EV [59].

After the administration of OMVs isolated from the feces of AD patients to C57BL/6J mice, Wei et al. observed increased BBB permeability and activation of the GSK3β pathway (glycogen synthase kinase 3β) [56]. This resulted in hyperphosphorylation of the Tau protein, which caused destabilization of microtubules and the formation of intracellular neurofibrillary tangles. This, in turn, disrupted intraaxonal transport and resulted in the death of neurons. OMVs were also shown to activate astrocytes and microglia, and cause the increased expression of pro-inflammatory cytokines (NFκB, interleukin-1β and TNF-α). These changes may lead to the cognitive disorders observed in AD patients [60]. Since OMVs were administered directly into the bloodstream, this study does not fully imitate the pathogenesis process, but it clearly indicates the ability of vesicles to cross the BBB. Wei and colleagues also found significant differences between OMVs isolated from AD patients and those from healthy subjects. AD-OMV displayed highly elevated levels of aspartate, L-aspartate, imidazole-4-acetate and L-glutamate. Differences in the levels of metabolites such as arachidic acid, prostaglandin G2 and leukotriene B4 were also identified. The significance of this variation has yet to be established [60].

The microorganisms that have attracted considerable attention in the context of neurodegenerative diseases are bacteria responsible for periodontitis. This is an inflammatory disease in which the structures supporting the teeth are damaged. The anaerobic *Porphyromonas gingivalis* is considered the key pathogen responsible for its development by generating dysbiosis and dysregulating the host’s innate immune response. *P. gingivalis* produces outer membrane vesicles that contain many virulence factors, including lipopolysaccharide (LPS) and gingipains—enzymes with proteolytic activity [61,62].

Gingipains have a very strong influence on the functioning of the host’s immune system. They have been shown to degrade antimicrobial peptides, complement components, antibodies, cytokines and their receptors, and inactivate intracellular signaling pathways. These enzymes are also responsible for breaking connections between the endothelial cells that line the vasculature, so are likely to render OMVs capable of crossing the BBB [63]. Levels of gingipains in OMVs are three to five times higher than in the *P. gingivalis* cells producing them in this form [64]. These proteases pass beyond the bacterial biofilm located in the gingival pocket and can penetrate the other tissues, which significantly increases their range of action. Using small-molecule inhibitors targeting gingipains, Dominy and co-workers were able to block the production of Ab1-42, a component of amyloid plaques, which reduced nervous system inflammation in the hippocampus. These authors postulated that after entering the brain, *P. gingivalis* OMVs activate the NLRP3 inflammasome, and as a result caspase 1 enhances the production of pro-inflammatory cytokines (IL-1β and IL-18), high levels of which may lead to pyroptotic neuronal death [55]. This is consistent with the observation that OMV-associated PAMPs (e.g., LPS) can be sensed intracellularly and can activate the inflammasome cascade and pyroptosis [65].

Thus, membrane vesicles do not always carry exotoxins, but are able to cause damage by inducing an inflammatory response in the host organism.

### 3.3. Effects on Cells of the Immune System

The ability to produce membrane vesicles is not restricted to pathogenic bacteria. Commensal microorganisms also secrete this form of vector to transfer effectors and other types of information over long distances. The richest bacterial community in mammals is found in the gut, which is also one of the most important parts of the innate immune system. The cells lining the mucosal surface are the first line of contact between host and microbiome, but not the only one. A variety of immune cells, i.e., monocytes, dendritic cells, neutrophils and lymphocytes, are located under the semi-permeable epithelium. These cells are able to recognize foreign elements, react and control the immune response against pathogens. Moreover, every cell type can sense and respond to bacterial effectors by increasing, decreasing or switching the immune response via a few major mechanisms [66].

One of the most important features of MVs is that they are rich in antigens. Almost any type of antigen can react with receptors on human cells and become a factor in the activation of an immune response. The first effect of MVs on a mammalian host is the activation of the pro-inflammatory reaction. *H. pylori* is a human pathogen living in the stomach near the mucosal surface. OMVs produced by *H. pylori* can increase the level of the pro-inflammatory cytokine interleukin 8 (IL-8) in stomach tissue and the severity of the pro-inflammatory reaction depends on the dose of OMVs [67]. The intestinal mucosal membrane is not the only internal microbial barrier in the human organism; parts of the respiratory system (bronchi and pulmonary alveoli) also participate in anti-pathogen reactions. Cystic fibrosis is a serious genetic disease that increases the risk of chronic infection. Thick mucus, accumulating in the respiratory tract of a person with cystic fibrosis, is an ideal environment for bacterial growth. A species commonly encountered in the respiratory system mucosa is *P. aeruginosa*. In response to OMVs of *P. aeruginosa*, primary bronchial epithelial cells as well as immune cells were shown to secrete IL-8 [68]. The reaction with the OMVs of *L. pneumophila* appears to be more complicated. Following incubation with a suspension of *L. pneumophila* OMVs, human bronchial epithelial cells produced several immunomodulatory proteins: CCL-2 (CC-chemokine ligand 2), IL-7, IL-8, IL-13, G-CSF (granulocyte colony-stimulating factor) and IFN-β (interferon-β) [69].

The surface and the insides of OMVs are rich in PAMPs such as proteins, lipids, DNA, RNA, LPS and peptidoglycan. Their presence and interaction with pattern recognition receptors (PRRs) initiate signaling cascades and the production of pro-inflammatory effectors like cytokines and chemokines, but also antimicrobial peptides, as the first lines of immune defense. Although the cargo of OMVs differs among bacterial species, the reactions of epithelial and immune cells to them are similar. There are a few canonical antigens that are recognized similarly. For example, toll-like receptor 4 (TLR4) is a basic receptor that recognizes LPS as a marker of infection. OMVs and OIMVs present LPS on their surface and initiate an immune reaction. TLR4 complexes are also presented on the luminal surface of epithelial cells and constitute the first line of immune defense. Activation of TLR4 is the initiating step of the NF-κB signaling pathway and the production of pro-inflammatory cytokines, which leads to the activation of the innate immune system [70].

Except for pro-inflammatory production, there are other host reactions triggered by the recognition of pathogen MVs. One of the most important responses is secretion of antimicrobial peptides (APs), a class of small (10–50 amino acids) stable peptides produced by many phylogenetic groups. Almost all APs are negatively charged on their outer surface, which allows them to interact with the surface of pathogens (gram-negative and gram-positive bacteria, enveloped viruses, fungi) and also cancer cells. There are several models for the mechanism of AP action but the most popular is integration of the peptide within the membrane of the pathogen, thereby increasing its permeability [71].

MVs are able to modulate (increase, decrease or change) basic functions of neutrophils including pathogen recognition, secretion of pro- and anti-inflammatory signals, and killing of foreign cells. The mechanism of action and its effects are determined by the cargo of the MV, which differ according to the bacterial species and the physiological conditions. The OMVs of uropathogenic *E. coli* (UPEC) contain a special virulence factor—cytotoxic necrotizing factor type 1 (CNF-1). This factor decreases phagocytosis and the regulatory abilities of neutrophils [72]. One of the most spectacular weapons that neutrophils can deploy against pathogens are neutrophil extracellular traps (NETs). NETs contain a net-like mixture of DNA from cell nuclei and enzymes with antimicrobial properties. Non-specific DNases originating from lysed bacterial cells that accumulate on the surface of MVs can destroy NETs by DNA hydrolysis [73]. Consequently, along with the other contents of MVs, these enzymes are thought to be important non-canonical virulence factors.

Besides their basic pro-inflammatory properties (due to the reaction with PRRs), MVs can also decrease the macrophage response. One example is the OMVs of *H. pylori* which induce production of the anti-inflammatory effector IL-10 to promote bacterial survival [74]. OMVs of *Brucella abortus* decrease the immune response by interaction with Toll-like receptors TLR2, TLR4 and TLR5. Without this important system of PRRs, the recognition of and efficient reaction against pathogens is impossible [75].

MVs and their factors are also able to decrease human adaptive immunity. OMVs of *H. pylori* are a well-studied example of this group of virulence factors. They suppress the activity of T cells by increasing the quantity of active caspase-3 and caspase-7 in these cells, causing apoptotic death [74]. Similarly OMVs of *N. meningitidis* contain Opa proteins which inhibit T cell activation and proliferation. T cells are the first step of the adaptive reaction and their suppression may be a universal strategy to create a safe area for chronic infection [76].

*Moraxella catarrhalis* is a Gram-negative bacterium isolated from the mucosal surface of the respiratory system. It produces OMVs containing Moraxella IgD-binding protein (MID), which is a superantigen. This protein causes the internalization and deactivation of BCR—universal receptors of B cells. Consequently, the functioning of a core component of the humoral immune response is reduced [77]. *Haemophilus influenzae* uses a different pro-activating way to blunt the immune response. B cells stimulated by OMVs from this bacterium are activated, proliferate, and produce polyclonal antibodies (IgM and IgG), but these antibodies do not recognize *H. influenzae* PRRs. *Neisseria lactamica*, a commensal species of the nasopharynx in young children, produces OMVs that induce non-specific B cell proliferation [78].

The pro- and anti-inflammatory effects of MVs are the most important elements in chronic infection. Two bacterial species associated with long-term inflammatory disease, *P. gingivalis* and *H. pylori*, produce OMVs rich in effectors. In addition to the typical effects in the infected tissue, these vesicles are involved in multi-organ and long-distance effects, such as autoimmune responses, arteriosclerosis, and other cardio-vascular diseases [79].

As mentioned previously, the ability to produce MVs is not unique to pathogenic bacteria. The anaerobic Gram-negative commensal bacterium *Bacteroides fragilis* secretes polysaccharide-A (PSA). This factor contacts cells of the host’s immune system in association with OMVs. It reduces the immune response by decreasing the production of IL-17 (interleukin with pro-inflammatory features) and its secretion in gut tissue. As a result, not only do these MVs protect autochthonous bacterial sub-populations of the intestine, they also prevent excessive immune responses against food antigens [80].

## 4. Conclusions

The molecular understanding of bacterial virulence factors is an important challenge for microbiologists. Modern techniques have enabled the discovery of novel mechanisms that sometimes surprise researchers with their universality. This is the case for membrane vesicles, which play important roles in the interactions of bacteria with cells of other organisms. MVs are not only a new type of secretion system, their great variety of structure and function, action at a distance, and stability in the host system make them an important weapon in the bacterial arsenal. Examples of the best characterized bacterial virulence factors associated with MVs are presented below in Table 1.

## Figures and Tables

**Table 1:** Examples of bacteria producing membrane vesicles and active factors discovered inside/outside MVs.

**Bacterial Species (Gram-Negative)**	**Active Factors**	**Reference**
*Acholeplasma laidlawii* PG8	adhesins/invasins—enable tight physical contact between bacterium and host cell ABC transporting complexes hydrolases: proteases, nucleases, and glycosylases metallo-β-lactamase	[81]
*Acinetobacter baumannii*	AmpC—β-lactamase OmpA—porin with potential cytotoxic features	[82]
*Actinobacillus pleuropneumoniae*	Apx—exotoxin with cytolytic features	[83]
*Aggregatibacter actinomycetemcomitans*	leucotoxin (Ltx)—induces lysis of monocytes and neutrophils AbOmpA—porin that enables transport of soluble substances in MV lumen across membrane	[84]
*Bartonella henselae*	HbpC—protein accumulating hemin; hemin sequestration protects bacteria from toxic concentrations of this porphyrin	[85]
*Borrelia burgdorferi*	enolases—enzymes cleave plasminogen to plasmin active form OspA/B/D—lipoprotein; promotes adhesion of OMVs to host cells (especially cells of endothelium)	[86,87]
*Burkholderia cepacia*	spreading factors—non-specific lipases and proteases (including metallo-proteases)	[88]
*Campylobacter jejuni*	CDT—three-component genotoxin (CdtA/B/C) with endonuclease features, stops cell cycle at G2/M phase check-point	[42]
*Coxiella burnetti*	periplasmic effector proteins	[89]
*Escherichia coli* K1	OmpA—interaction with Ecgp receptor on surface of brain microvascular endothelium leads to cell invasion; may also act in trans to promote cell invasion by other bacterial species K1 antigen—polysaccharide antigen from cell envelope, linear polymer of NeuNac TLR ligands—flagellin, lipoproteins, poly-CpG DNA strands	[90]
*Escherichia coli* O157: H7 *Shigella dysenteriae*	Shiga toxin (Stx1/2)—toxin from AB5 group with RNA-N-glycosylases activity; stops eukaryotic translation	[91]
enterotoxic *E. coli* (ETEC)	thermolabile toxin (LT)—activates adenylate cyclase to elevate cAMP levels which disturbs water management of host cell; form linked to OMVs may also be non-febrile adhesin	[92]
enterohemorrhagic *E. coli* (EHEC)	ClyA—pore-forming cytolysin; reducing environment of OMV lumen promotes ClyA oligomerization to produce active complex HlyA—alpha-hemolysin; damages enterocyte mitochondrial membranes	[93]
extraintestinal pathogenic *E. coli* (ExPEC)	HlyA—alpha-hemolysin CNF1—cell necrosis factor	[94]
*Haemophilus influenzae* type B (Hib)	LPS and other strong surface antigens proteins that assist in process of biofilm formation	[95]
*Legionella pneumophila*	Map—acidic phosphatase ProA1—metallo-protease LasB—elastase legionaminic acid—component of LPS O-antigen inhibitors of phagosome-lysosome fusion	[96]
*Moraxella catarrhalis*	MID—protein linking IgD, superantigen UspA1/2—blocks C3 protein of complement system Bro1/2—beta-lactamase	[97]
*Neisseria meningitidis* serogroup B	PorA—main surface antigen of OMVs; potential component of future vaccine LpxL1—strong adjuvant	[76]
*Porphyromonas gingivalis*	gingipains—non-specific proteases degrading elements of host’s tissue and cytokines HmuY—lipoprotein accumulating heme; assists biofilm formation process factors assisting in co-localization with *Treponema denticola*	[98]
*Salmonella enterica*	SopB—protects SCV (*Salmonella*-containing vacuoles) from degradation by reorganization of actin cytoskeleton SipC—protein assisting in cell invasion process SopA—ubiquitin ligase (E3) disturbing ubiquitin pathway of host cell FljB—flagellin, strong antigen SopE2—guanine nucleotide exchange factor (GEF); by catalysing exchange GDP → GTP disturbs function of Rho-protein family GTPases controlling dynamics of host cell cytoskeleton, which leads to membrane surface deformation and assists invasion process PagK1/2—exact function still unknown; probably assists bacterial proliferation inside SCV SrfN—promotes bacterial survival inside macrophages	[99]
*Shigella flexneri*	IpaD—controls cell invasion process IutA—iron-siderophore receptor	[100]
*Treponema denticola*	dentilisin—protease	[101]
*Vibrio cholerae*	cholera toxin (CTx)—AB_5_ group toxin; disturbs ion-transfer across cell membranes and water management	[27]
*Yersinia pestis*	Ail—surface adhesin; promotes contact with host cells Pla—extracellular protease; activator of plasminogen Caf1—fimbrial antigen F1; main component of OMVs	[102]
**Bacterial Species (Gram-Positive)**	**Active Factors**	**Citations**
*Bacillus anthracis*	anthrolysin (ALO)—cholesterol-dependent cytolysin lethal factor (LF)—zinc-protease; hydrolyses several MAPK-kinases (MAPKK), causes disturbance of signalling pathways and cell death edema factor (ED)—calmodulin- and Ca^2+^-dependent adenylate cyclase; induces uncontrolled increase in cAMP concentration in phagocytic cells thus depleting ATP reserves	[12]
*Clostridium perfringens*	N-acetylglucosamine—important pro-inflammatory factor	[103]
*Enteroccoccus faecium*	phospholipids; reduce antibacterial activity of the antibiotic daptomycin SdrD—collagen-binding protein PavA—fibronectin-binding protein AtlA—autolysin; assists in biofilm formation process Acm—MSCRAMM (microbial surface components recognizing adhesive matrix molecules) group adhesin; binds collagen Fnm—fibronectin-binding adhesin PsaA—lipoprotein; potential component of future vaccine	[104]
*Mycobacterium tuberculosis*	LpqH—lipoprotein; assists in transport processes MPB83—highly immunogenic glycoprotein LprA—lipoprotein; strong TLR2 agonist PSTS3—component of ABC transport system connected with phosphorus ion import lipoarabinomannan (LAM)—surface glycolipid with anti-ROS features mycobactin—surface Fe^3+^-siderophore	[105]
*Propionibacterium acnes*	factors activating TLR2-dependent inflammation	[106]
*Streptococcus mutans*	eDNA—important biofilm component glucosyltransferases (GtfB/C/D)—produce adhesive extracellular polysaccharides from sucrose substrate lipoteichoic acid (LTA)—surface antigen; important in adsorption process in biofilm formation	[107]
*Streptococcus pneumoniae*	TatD—non-specific DNase enabling degradation of NETs (DNA nets associating with proteins with antimicrobial activities: LL37, myeloperoxidase, neutrophil elastase) EndA—non-specific DNase located on surface of MVs PspC—H factor-binding protein; blocks alternative complement pathway pneumolysin (Ply)—exotoxin with cytolytic features PsaA—adhesin; strong surface antigen SatA—ABC-type transporter; surface antigen AmiA—peptide-binding protein; assists in active transport MalX—maltose and maltodextrin-binding protein PnrA—ABC-type nucleoside transporter spr1909—penicillin-binding protein	[108]

## Data Availability

Not applicable.
